# Permissivity of primary hepatocytes and hepatoma cell lines to support hepatitis C virus infection

**DOI:** 10.1099/vir.0.000085

**Published:** 2015-06

**Authors:** Garrick K. Wilson, Michelle J. Farquhar, Luke Meredith, Anil Dhawan, Ragai Mitry, Peter Balfe, Jane A. McKeating

**Affiliations:** ^1^​ Institute for Biomedical Research and NIHR Liver Biomedical Research Unit, Centre for Human Virology, Viral Hepatitis Laboratory, University of Birmingham, Birmingham B15 2TT, UK; ^2^​ Institute of Liver Studies, Kings College Hospital and Kings College London, London SE5 9RS, UK

## Abstract

The major cell type supporting hepatitis C virus (HCV) infection is the hepatocyte; however, most reports studying viral entry and replication utilize transformed hepatoma cell lines. We demonstrate that HCV pseudoparticles (HCVpp) infect primary hepatocytes with comparable rates to hepatoma cells, demonstrating the limited variability in donor hepatocytes to support HCV receptor-glycoprotein-dependent entry. In contrast, we observed a 2-log range in viral replication between the same donor hepatocytes. We noted that cell proliferation augments pseudoparticle reporter activity and arresting hepatoma cells yields comparable levels of infection to hepatocytes. This study demonstrates comparable rates of HCVpp entry into primary hepatocytes and hepatoma cells, validating the use of transformed cells as a model system to study HCV entry.

Hepatitis C virus (HCV) affects 3 % of the world’s population and is a leading cause of chronic liver disease, including fibrosis, cirrhosis and hepatocellular carcinoma (Alter, 2007). The recent availability of direct-acting antiviral agents has led to a significant improvement in patient response rates (Scheel & Rice, 2013). However, the cost of such treatments will be prohibitive in many parts of the world, highlighting the need for immune-based therapies for treating chronic hepatitis C and the development of a preventive vaccine. HCV primarily infects hepatocytes in the liver that are highly differentiated and arrested in the G1 stage of the cell cycle (Farquhar & McKeating, 2008; Taub, 2004). HCV initiates infection by attaching to molecules or receptors at the cell surface and current evidence supports an essential role for scavenger receptor class B member I (SR-BI), tetraspanin CD81 and tight-junction proteins claudin-1 and occludin in clathrin-dependent particle endocytosis (Meredith *et al.*, 2012; Ploss & Evans, 2012). Additional accessory factors include low-density lipoprotein receptor, epidermal growth factor receptor, Niemann–Pick C1-like cholesterol receptor and transferrin receptor (Baumert *et al.*, 2014). To date, much of our understanding of the viral life cycle derives from *in vitro* studies with lentiviral pseudotypes transiently expressing HCV-encoded glycoproteins (HCVpp) and infectious molecular clones based on the JFH-1 strain of virus (HCVcc) infecting transformed hepatoma cell lines (Farquhar & McKeating, 2008; Wilson & Stamataki, 2012). In contrast, there are limited reports of studies of HCV infection of primary hepatocytes (PHHs) (Fofana *et al.*, 2010; Podevin *et al.*, 2010; Régeard *et al.*, 2008) and importantly a lack of studies comparing their permissivity to hepatoma cell lines.

First, we optimized the protocol for infecting PHHs with HCVpp and HCVcc strain SA13/JFH. The former allows the measurement of HCV glycoprotein-dependent entry. Cells were infected 1, 2 or 4 days post-seeding with luciferase-reporter pseudoparticles expressing HCV strain H77 genotype 1a glycoproteins (HCVpp-H77) or control vesicular stomatitis virus glycoprotein (VSV-Gpp) and HCVcc in the presence or absence of monoclonal antibodies targeting SR-BI or CD81. Infection was assessed 72 h later by measuring luciferase or viral RNA, respectively (Fig. 1a–c). HCVpp and HCVcc infected PHHs optimally at 2 days post-seeding, consistent with increased expression of SR-BI and claudin-1 compared with day 1 cells (Fig. 1d–e). However, it is noteworthy that CD81, SR-BI, claudin-1 and occludin expression was optimal at 4 days post-plating, suggesting that other factors independent of receptor expression levels may regulate HCVpp entry. In contrast, VSV-Gpp infected PHHs at all times post-seeding with comparable luciferase activity (Fig. 1b). Anti-CD81 and anti-SR-BI antibodies inhibited infection, confirming receptor-dependent virus entry (Fig. 1a). We noted similar IC_50_ values for both anti-receptor antibodies to neutralize HCVpp infection of PHHs and hepatoma cell lines (data not shown). Having determined the optimal time to infect PHHs after isolation from the liver, we compared the rate of HCVpp and VSVpp infection of PHHs with the permissive Huh-7.5 hepatoma cell line (Blight *et al.*, 2003; Schwarz *et al.*, 2009). Virus was allowed to infect target cells for increasing time periods up to 8 h, unbound virus was removed by washing and luciferase activity was measured after 72 h (Fig. 2). We observed comparable rates of HCVpp [4535±220 and 4728±345 relative light units (RLU) h^−1^] and VSV-Gpp (265 483±106 122 and 319 216±59 485 RLU h^−1^) infection of PHHs and Huh-7.5 cells, respectively.

**Fig. 1.**
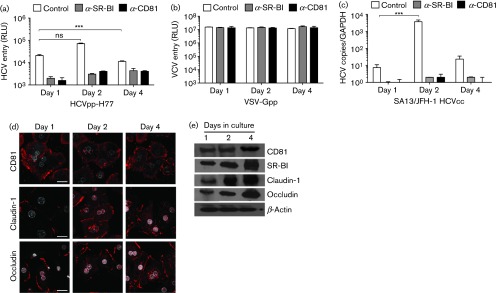
Optimizing HCV infection of primary human hepatocytes. (a, b) Human hepatocytes were isolated according to previously published protocols (Mitry, 2009) and maintained in Williams E medium supplemented with 10 % FBS/5 mM HEPES/insulin/dexamethasone. Hepatocytes were plated at 4×10^4^ cm^−2^ on collagen-coated plates and infected with HCVpp-H77 or VSV-Gpp prepared as previously described (Hsu *et al.*, 2003). Briefly 293T HEK cells were transfected with two plasmids, one encoding the HIV provirus expressing luciferase and the other encoding HCV E1E2, VSV-G or a no-envelope control. Supernatants were collected 48 h later, clarified, evaluated for HIV p24 content and used to infect target cells for 8 h with an inoculum of 1 ng p24 per well at 1, 2 and 4 days post-seeding. Virus was removed by washing and cultures were maintained at 37 °C for 72 h. At 72 h post-infection, cells were lysed and luciferase activity was measured using a luminometer (Lumat LB 90507). Cells were incubated with anti-SR-BI or anti-CD81 (2s131) monoclonal antibodies at 5 µg ml^−1^ prior to being inoculated with virus. Data are presented as relative light units (RLU) from which a no-envelope pseudotype control value has been subtracted, and are representative of three experiments. Error bars indicate sd from the mean (*n* = 3); ***P<0.001; ns denotes no statistical significance. (c) Cells were infected with HCVcc SA13/JFH as previously described (Jensen *et al.*, 2008). Virus-containing medium (1.8×10^6^ focus-forming units ml^−1^, approximate m.o.i. 0.01) was added to target cells for 8 h followed by washing and change of medium. Infection was realized 72 h later by quantitative reverse transcriptase PCR detection of the cellular viral RNA load using the Cells Direct kit (Life Technologies). Data are presented as HCV RNA copy number relative to GAPDH and are representative of three independent experiments. ***P<0.001. (d) Confocal microscopy images of HCV receptor molecules CD81 (2s131), claudin-1 and occludin (Life Technologies) in PHHs at 1, 2 or 4 days post-seeding on glass coverslips. Cells were methanol-fixed and permeabilized with saponin (Sigma) followed by incubation with respective antibodies (1 : 1000) for 1 h at room temperature in a PBS/BSA solution. Cells were washed three times before the addition of species-appropriate Alexa-594-conjugated secondary antibodies (1 : 1000) for 1 h at room temperature. After a further wash the nuclei were counterstained with DAPI and coverslips were mounted on glass slides using ProLong Gold antifade (Life Technologies). Scale bars represent 20 µm. (e) Western blot detection of HCV receptor molecules including SR-BI in PHHs lysed at 1, 2 or 4 days post-seeding using a previously published protocol (Farquhar *et al.*, 2012).

**Fig. 2.**
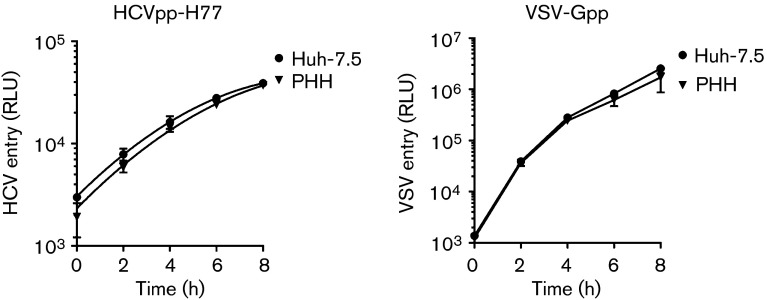
HCV entry kinetics. PHHs and Huh-7.5 cells were seeded at 4×10^4^ cm^−2^ on collagen-coated plates and 2 days post-plating were infected with HCVpp or VSV-Gpp. The inoculum was removed at 2 h intervals up to 8 h and cultures were maintained for 72 h. Data are presented as RLU from which a no-envelope pseudotype control value has been subtracted and are representative of three independent experiments. Error bars indicate sd from the mean (*n* = 3).

To assess the impact of donor variability on PHH permissivity we compared cells isolated under GLP conditions from five donors for their ability to support HCVpp and HCVcc infection. All PHH preparations showed comparable levels of HCVpp or VSV-G infection (Fig. 3a, b). Similar data were obtained with HCVpp expressing a variety of primary envelope glycoproteins (data not shown). As controls, we included two commonly used hepatoma cell lines, Huh-7.5 and Hep3B. We noted that the HCVpp-encoded luciferase values for PHHs were approximately 1-log lower compared with both hepatoma cells, suggesting a lower frequency of infected cells in the PHH population. The efficiency of the lentiviral promoter in the different cell types could in part explain the different luciferase signals. However, this is unlikely since the majority of donor primary and hepatoma cells showed comparable VSV-Gpp luciferase signals (Fig. 3b). In contrast to the pseudoparticle infectivity data, we noted significant variability in the ability of PHHs isolated from different donors to support replication of HCV strains JFH-1 and SA13/JFH (Fig. 3c), with levels of HCV RNA in PHHs at least 1-log lower than in Huh-7.5 cells (Fig. 3c).

**Fig. 3.**
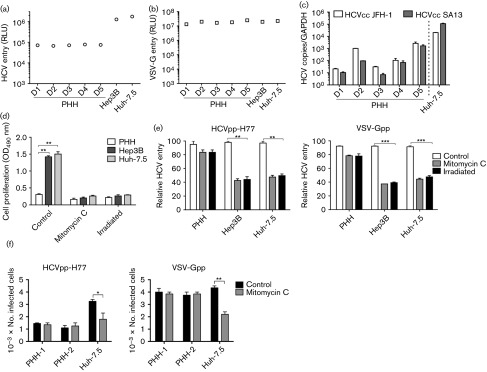
Cell proliferation augments pseudoparticle infection. PHHs, and Hep3B and Huh-7.5 cells were seeded at 4×10^4^ cm^−2^ on collagen-coated plates and 2 days post-plating were infected with HCVpp, VSV-Gpp (a, b) or HCV JFH-1 and SA13/JFH (c) virus for 8 h as detailed in Fig. 1. Data are presented as RLU from which a no-envelope pseudotype control value has been subtracted or as HCV RNA copy number relative to GAPDH and are representative of three independent experiments. (d) Mitomycin C and γ-irradiation block cell growth. Cells were treated with mitomycin C (10 µg ml^−1^, overnight) or γ-irradiated (32 mSv, 30 min) and proliferation was measured using an MTT assay (Promega) as per the manufacturer’s instructions; data are presented as OD_450_. (e) Mitomycin C or γ-irradiated cells were infected with HCVpp-H77 or VSV-Gpp. Cultures were maintained at 37 °C for 72 h and luciferase activity was measured as described above. Data are presented relative to untreated cells. (f) Control and mitomycin C-treated PHHs (donor 2) and Huh-7.5 cells were studied for their ability to support HCVpp-H77 and VSV-G pseudoviruses expressing green fluorescent protein (GFP) reporter. GFP-expressing cells were detected 72 h post-infection by flow cytometry. *P<0.05, **P<0.01, ***P<0.001 (*t*-test). Error bars indicate sd from the mean (*n* = 3).

It is widely accepted that pseudoparticle reporter signals reflect differences in glycoprotein-dependent entry, and luciferase values are commonly used as a read-out in antibody-based neutralization assays and for measuring strain-dependent differences in virus entry. In the liver, hepatocytes are largely quiescent and PHHs show minimal proliferative capacity *in vitro* (Taub, 2004). In contrast, hepatoma cells have a doubling time in the order of 18–24 h (Sainz *et al.*, 2012b). We hypothesized that proliferation of an infected cell may result in progeny daughter cells bearing the reporter gene, resulting in an increased luciferase signal that does not reflect the initial viral entry event per se. To ascertain whether cell proliferation modulates HCVpp and VSV-Gpp luciferase signals, PHHs and hepatoma cells were arrested with mitomycin C or γ-irradiation and evaluated for pseudoparticle infection. Mitomycin C and γ-irradiation limited hepatoma growth and had no discernible effect on PHH proliferation or viability (Fig. 3d). Both treatments had minimal effect on HCVpp or VSV-Gpp entry into PHHs, consistent with the non-dividing nature of these cells. In contrast, both treatments significantly reduced HCVpp and VSV-Gpp infection of the hepatoma cell lines, resulting in comparable luciferase values to PHHs (Fig. 3e). To confirm our earlier model that Huh-7.5 proliferation affects the number of pseudoparticle-infected cells, we inoculated non-arrested and arrested PHHs and Huh-7.5 cells with HCVpp-H77 and VSV-Gpp bearing a GFP reporter. Mitomycin C reduced the number of HCVpp and VSV-Gpp-GFP-expressing Huh-7.5 cells but had no impact on the number of infected PHHs (Fig. 3f). Importantly, following mitomycin C treatment the numbers of HCVpp-infected PHHs and Huh-7.5 cells were comparable (Fig. 3f).

In summary, we have shown that PHHs from multiple donors support similar levels of HCVpp entry. In contrast, the same donor hepatocytes showed significant differences in their ability to support HCVcc replication, consistent with recent reports showing donor-dependent differences in innate signalling and type III interferon expression (Li & Lemon, 2013; Marukian *et al.*, 2011; Park *et al.*, 2012). This study raises an interesting question as to whether differences in the frequency of infected hepatocytes and viral RNA reported in chronically infected subjects (Liang *et al.*, 2009) are largely defined at the level of viral replication and innate immune signalling rather than virus entry. We report for the first time that hepatoma proliferation increases HCVpp reporter signals, demonstrating that luciferase values are not solely defined by the HCVpp entry process. This is particularly pertinent when comparing HCV entry into different cell types with varying growth rates or when evaluating the effect of antibodies, growth factors or kinase inhibitors with known effects on cell proliferation (Lupberger *et al.*, 2011; Marukian *et al.*, 2008; Sainz *et al.*, 2012a). In summary, growth-arrested hepatoma cell lines support comparable rates of HCVpp entry into primary hepatocytes, validating their use as a surrogate model system.
